# Expression and Role of the RNA‐Binding Protein, ZFP36L1, in Mouse T Follicular Helper Cell Differentiation and Function

**DOI:** 10.1002/eji.70260

**Published:** 2026-08-18

**Authors:** Helena A. Carslaw, Sam Woolliscroft, Emily M. Watson, Sarah E. Bell, Michelle A. Linterman, Martin Turner, Louise M. C. Webb

**Affiliations:** ^1^ Immunology Programme Babraham Institute Cambridge UK; ^2^ Kennedy Institute of Rheumatology University of Oxford Oxford UK; ^3^ MRC Laboratory of Molecular Biology, Francis Crick Avenue Cambridge Biomedical Campus Cambridge UK; ^4^ The Malaghan Institute of Medical Research Wellington New Zealand

**Keywords:** CD4+ T cells, germinal center, RNA‐binding proteins, T follicular helper cell

## Abstract

T follicular helper (Tfh) cells are critical for germinal centers (GC), the specialized microenvironment where long‐lived humoral immunity is generated in response to vaccination or infection. Within the GC, B cells engage with Tfh cells and elicit their help in the form of cytokines and cell‐surface co‐stimulator molecules. Tfh helper activity must be rapidly available in response to B cell engagement, yet the mechanisms controlling this helper activity remain poorly characterized. Post‐transcriptional regulation of mRNA decay and translation offers one way to rapidly and temporally tune Tfh cell activity. ZFP36L1, a member of the ZFP family of RNA‐binding proteins, is a candidate modulator of Tfh cell helper activity, as it controls cytokine production and responses in other T cell lineages, modulating their differentiation and function. We sought to determine if ZFP36L1 is also important for Tfh cell biology. In this study, we show expression of ZFP36L1 by Tfh cells. We selectively delete ZFP36L1 from Tfh cells and analyze the effect this has on the GCs. Surprisingly, we find that the GC response and affinity maturation are resilient to deletion of ZFP36L1 from Tfh cells.

## Graphical abstract

T follicular helper (Tfh) cells support germinal center (GC) B cells, generating long‐lived antibody‐secreting and memory B cells. RNA‐binding proteins likely fine‐tune Tfh function, but selective deletion of ZFP36L1 from Tfh cells alters neither their differentiation nor GC responses. This suggests further redundancy within the ZFP36 family.

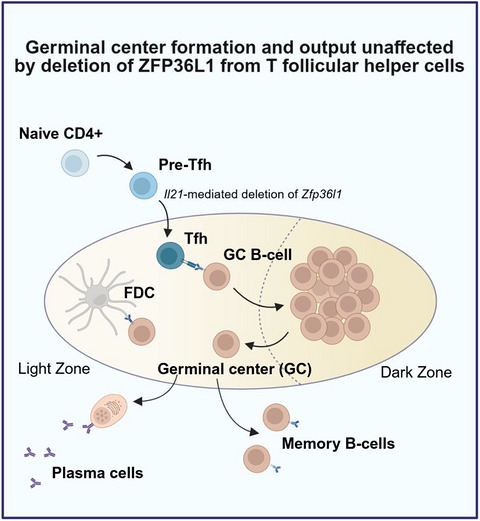

**Abbreviations**
GC: germinal center
RBP: RNA‐binding proteins
Tfh: T follicular helper cell
ZFP: zinc finger protein

## 1 Introduction

Germinal centers (GCs) form in response to vaccination or infection. They are pivotal for the generation of robust and effective immune responses, generating lifelong immunity via the formation of memory B cells and antibody‐secreting cells (ASCs) [1]. T follicular helper (Tfh) cells are a subset of CD4^+^ T cells essential for the development and function of GCs [2]. Tfh cells interact with antigen‐specific B cells both outside the GC at the T:B cell border, and within the light zone of the GC where they provide key helper signals via CD40 engagement and provision of cytokines. Rapid elicitation of Tfh cell help by engaged B cells is paramount for B cell entry into the GC and their subsequent rapid proliferation within it [1, 2, 3]. However, this help must be carefully controlled to prevent aberrant immune responses and to maintain the selective environment critical for ensuring affinity maturation of the B cell response [1].

RNA‐binding proteins (RBPs) are candidate regulators of Tfh cell activity, as they can provide the rapid response to stimuli required for T–B cell interactions within the GC. They orchestrate rapid cellular responses by eliciting post‐transcriptional control of cytokines and other immune mediators [4]. By controlling RNA translation and/or mediating its decay, RBPs play critical roles in lymphocyte biology both in terms of development and function [5]. Thousands of RBPs have been annotated in T cells [6, 7, 8], over a hundred of which have been shown to bind to the untranslated region (UTR) of IFN‐γ, IL‐2, and IL‐10 mRNAs in human T cells, suggesting the potential for exquisite time/stimuli‐dependent control of protein production  [10].

Amongst the RBPs that interact with cytokine mRNAs is the zinc finger protein (ZFP)36 family, which is comprised of ZFP36, ZFP36L1, and ZFP36L2. They are recognized as important for lymphocyte function, mediating their effects via regulation of cytokine mRNAs and other transcripts encoding transcription factors, cell cycle and metabolic regulators [5]. They often play complex and overlapping roles. Whilst some responses of lymphocytes show unique dependencies on individual ZFP family members, others show redundancy [9, 10, 11]. For example, deletion of ZFP36L1 from human T cells results in increased half‐life of cytokine mRNA, and this is attributed to its ability to destabilize mRNA [12], whilst in memory T cells ZFP36L2 suppresses the translation of IFN‐γ mRNA [13]. However, deletion of both ZFP36L1 and ZFP36L2 in CD8^+^ T cells results in increased IFN‐γ production that is not seen with deletion of either ZFP family member alone [14]. In CD8+ T cells and T regulatory (Treg) cells, ZFP36L1 and ZFP36L2 are important for controlling responses to IL‐2 [15, 16]. In B cells, ZFP36L1 maintains the marginal zone B cell phenotype [17] and regulates plasma cell homing [18] but is dispensable for the GC reaction due to redundancy with ZFP36L2 [19]. Although the ZFP family participates in a multitude of lymphocyte functions, their role in Tfh cells remains unknown.

Tfh cells share transcriptional signatures with other T‐helper subsets, and until recently, it was not possible to specifically manipulate these cells in vivo via Cre‐directed targeting of genes. We recently repurposed an *Il21cre* fate‐mapping mouse to specifically target precursor‐Tfh, Tfh, and GC‐Tfh cells [20]. IL‐21 is the canonical cytokine produced by Tfh cells and plays a critical role in driving the GC response. Its expression is upregulated following cognate interactions with B cells. *Il21* gene expression emerges early during Tfh cell differentiation, first evident in a subset of precursor‐Tfh cells [20]. Here, we use this *Il21cre* fate‐mapping system to specifically delete *Zfp36l1* from Tfh cells, allowing us to assess its role in Tfh cell development and function. We show that despite expression of ZFP36L1 in Tfh cells, its loss does not affect GC formation or output, suggesting there may be redundancy in the ZFP family for Tfh cell function.

## 2 Results and Discussion

### 2.1 ZFP36L1 Expression During Tfh Cell Differentiation

**FIGURE 1:**
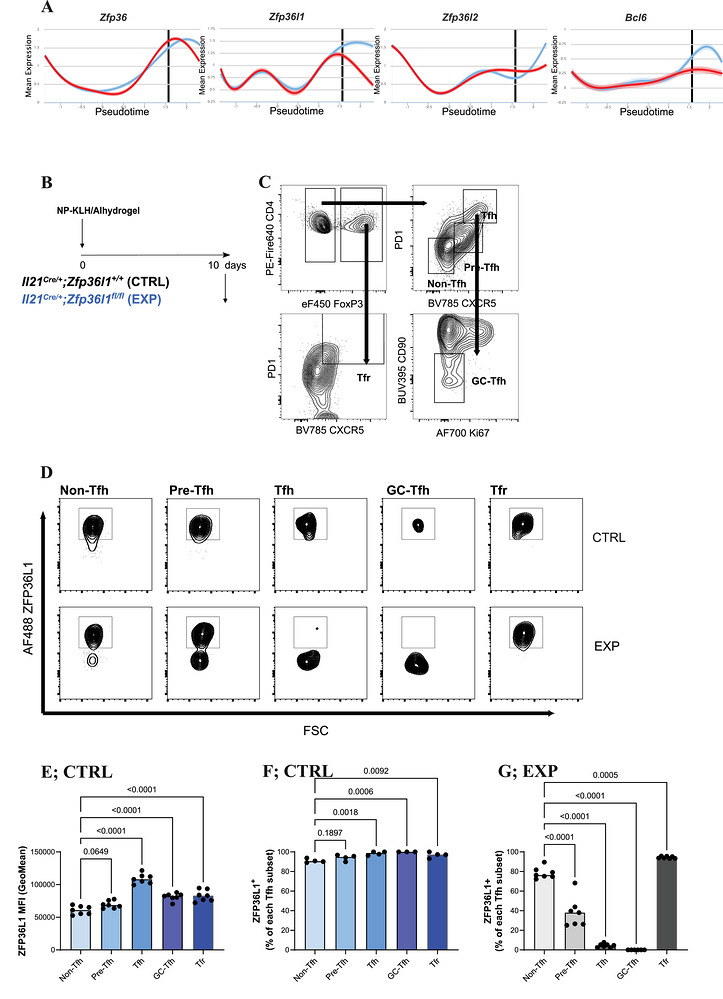
ZFP36L1 expression during Tfh cell differentiation. (A) Pseudotime analysis of *Zfp36*, *Zfp36l1*, *Zfp36l2*, and *Bcl6* expression by antigen‐specific TCR transgenic PbTII CD4^+^ cells following *P. chaubaudi* infection of mice. Relative expression levels of each gene in a single Tfh (blue line) or Th1 (red line) cells relative to pseudotime. (B) Experimental scheme for (C–G). *Il21^+/Cre^;Zfp36l1^+/+^* (CTRL) and *Il21^+/Cre^;Zfp36l1^fl/fl^* (EXP) mice were immunized subcutaneously with NP‐KLH/Alhydrogel and draining inguinal lymph nodes (iLNs) harvested 10 days later. (C) Gating strategy used for (E–G). (D) Representative flow cytometry plots showing staining for ZFP36L1 in different Tfh subsets following 3 h restimulation ex vivo for EXP or CTRL mice. (E) Bar graph showing expression levels of ZFP36L1 in different Tfh subsets as determined by flow cytometry for CTRL mice. (F,G) Bar graphs showing the percentage of cells expressing ZFP36L1 in CTRL (F) and EXP (G) mice. Bar graphs show medians, with each symbol representing an individual mouse. Data are representative of 2 independent experiments with 4–8 mice per group. A two‐way ANOVA was performed to determine statistical significance (*p*‐values shown on graphs).

Differentiation of naïve CD4^+^ T cells into Tfh cells occurs via a multistep process dependent on cognate interactions with antigen‐presenting cells (APCs) [21]. Naïve CD4^+^ T cells are first stimulated by APCs, resulting in their activation and formation of precursor Tfh cells (pre‐Tfh). Expression of CXCR5 and EBI2 by pre‐Tfh cells enables their migration and relocation to the interfollicular area between the T and B cell zones of the draining lymph node (LN; [21]). CD4^+^ T cells have a high degree of plasticity and pass through a bipotent Th1/Tfh precursor state, with subsequent engagement with B cells driving Tfh cell differentiation [22]. A subset of Tfh cells then enter the GC (GC‐Tfh), where they directly interact with GC B cells [23], driving the proliferation and affinity maturation of GC B cells [1, 2, 3]. We first sought to examine expression of the *Zfp36, Zfp36l1*, and *Zfp36l2* transcripts during these distinct phases of Tfh cell differentiation. Using published data from a model of Tfh and Th1 cell bifurcation following *Plasmodium chabaudi* infection [24], we examined pseudotime analysis of *Zfp36, Zfp36l1*, and *Zfp36l2* expression by antigen‐specific TCR transgenic PbTII CD4^+^ cells. This analysis showed that Tfh cells express *Zfp36, Zfp36l1*, and *Zfp36l2* mRNA and that expression is retained after the Th1/Tfh bifurcation point. Interestingly, *Zfp36l1* transcript expression closely mirrored that of the Tfh‐defining transcription factor, *Bcl6* (Figure 1A), suggesting a role for ZFP36L1 in Tfh differentiation/function. To determine if ZFP36L1 protein expression also accumulated in Tfh cells, we used intracellular staining of CD4^+^ T cells following immunization with a T‐dependent antigen, NP‐KLH. Using well‐defined markers for non‐Tfh, pre‐Tfh, Tfh, GC‐Tfh (GC‐resident Tfh), and T follicular regulatory (Tfr) cells [20], we determined ZFP36L1 expression during different stages of Tfh cell differentiation. Lymphocytes were harvested from the inguinal (draining) LN (iLN) within the peak of the GC response (days 9–11) after subcutaneous immunization with NP‐KLH/Alhydrogel (a time point where all Tfh cell subsets would be present), and ZFP36L1 protein expression was determined (Figure 1B). Non‐Tfh, pre‐Tfh, Tfh, GC‐Tfh, and Tfr cells were gated as shown (Figure 1C), and both the percentage of cells expressing ZFP36L1 and the levels of ZFP36L1 expression (MFI) were determined (Figure 1D–F). ZFP36L1 expression was seen in >90% of non‐Tfh, pre‐Tfh, Tfh, GC‐Tfh, and Tfr cells (Figure 1D–F). Although there were only incremental increases in the proportion of ZFP36L1^+^ cells in each subset during Tfh differentiation (Figure 1F), the amount of ZFP36L1 peaked at the Tfh stage of differentiation (Figure 1E) with an MFI nearly 2‐fold greater than that of pre‐Tfh and non‐Tfh cells. Thus, the majority of activated CD4^+^ T cells express ZFP36L1 protein following immunization, and ZFP36L1 expression is retained throughout the stages of Tfh cell differentiation, peaking before they enter the GC. This suggests ZFP36L1 could play a role in the development and/or biology of the GC response.

We examined ZFP36L1 expression in a strain of mice designed to delete *Zfp36l1* in Tfh cells (Figure 1D,G). *Il21*‐fate mapped (*Il21*‐fm) mice enable Cre expression in Tfh cells, GC‐Tfh cells, and a proportion of pre‐Tfh cells [20]. Importantly, *Il21*‐fate mapping spares suppressive Tfr cells, which function to balance Tfh cell helper function. In *Il21^+/Cre^;Zfp36l1^fl/fl^* mice, we observed loss of ZFP36L1 protein from Tfh cells, GC‐Tfh cells, and >50% of pre‐Tfh cells, but it remained present in Tfr cells (Figure 1D,G). The *Il21^+/Cre^;Zfp36l1^fl/fl^* mice thereby provided a valuable tool to investigate the function of ZFP36L1 in Tfh cell differentiation and biology.

### 2.2 Tfh Differentiation and Function in the Absence of ZFP36L1

**FIGURE 2:**
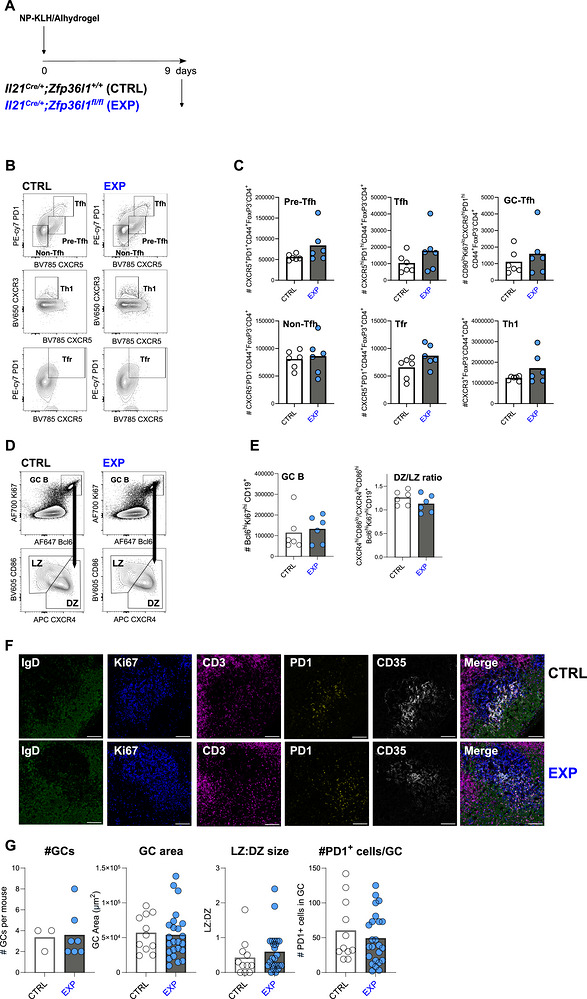
Tfh differentiation and function in the absence of ZFP36L1. (A) Experimental scheme for (B–E). *Il21^+/Cre^;Zfp36l1^+/+^* (CTRL) and *Il21^+/Cre^;Zfp36l1^fl/fl^* (EXP) mice were immunized subcutaneously with NP‐KLH/Alhydrogel and draining inguinal lymph nodes (iLNs) harvested 9 days later. (B) Representative flow cytometry plots showing Tfh subsets, Th1 cells, and Tfr cells in draining iLNs 9 days post‐immunization (d.p.i.). (C) Bar graphs showing the number of different T cell populations in CTRL (white circles) and EXP (blue circles) mice. (D) Representative flow cytometry plots showing germinal center (GC) B cells and light zone (LZ) and dark zone (DZ) GC B cells for CTRL and EXP mice. (E) Bar graphs showing the number of GC B cells and the ratio of DZ/LZ GC B cells in draining iLNs 9 d.p.i. in CTRL (white circles) and EXP (blue circles) mice. Data are representative of two independent experiments with 4–8 mice per group. (F) Representative confocal images of GCs from CTRL and EXP mice 9 d.p.i. IgD = green Ki67 = blue, CD3 = magenta, CD35 = white, PD1 = yellow). Scale bar = 100 µm. GC area is defined as the combined area of Ki67+ area and CD35+ area. (G) Bar graphs showing the quantification of GC number, size, and number of PD1+ cells in GCs from confocal images in CTRL (white circles) and EXP (blue circles) mice. Bar graphs show median values, with each symbol representing an individual mouse (for # GCs/mouse) or each symbol representing a GC. A two‐tailed, unpaired Mann–Whitney *U* test was performed to determine statistical significance; statistics were omitted from graphs if the *p*‐value was not significant.

Using *Il21*‐fm mice to specifically delete *Zfp36l1* from Tfh, GC‐Tfh cells, and a proportion of pre‐Tfh cells, we first analyzed Tfh cell differentiation by flow cytometry. Control (CTRL; *Il21^+/Cre^;Zfp36l1^+/+^)* or experimental (EXP; *Il21^+/Cre^;Zfp36l1^fl/fl^*) mice were immunized on both hind flanks with NP‐KLH/Alhydrogel and iLNs harvested for flow cytometry and imaging within the peak of the GC response (days 9–11; Figure 2A). Flow cytometry analysis showed normal Tfh and Tfr cell differentiation (Figure 2B,C) with no perturbations in Tfh or GC‐Tfh cells despite lacking ZFP36L1. Enumeration of the different Tfh cell subsets (non‐Tfh, pre‐Tfh, Tfh, GC‐Tfh, Tfr) confirmed this (Figure 2C). In addition, there was no difference observed in CXCR3^+^ Th1 cells (Figure 2B,C). Together, this shows that loss of ZFP36L1 does not affect Tfh cell differentiation or Th1/Tfh bifurcation.

Since the main function of Tfh cells is to provide help to B cells responding to antigen, it was important to determine if ZFP36L1 played a role in Tfh cell function. In T‐dependent responses, Tfh cells are critical for the formation and maintenance of the GC, mainly due to their provision of helper signals that drive GC B cell proliferation [2, 3]. To assess the role of ZFP36L1 in Tfh cell activity, we first examined GC formation in *Il21^+/Cre^;Zfp36l1^fl/fl^* (EXP) mice, comparing their responses to littermate controls (*Il21^+/Cre^;Zfp361^+/+^*; CTRL). As shown, GC B cells formed in *Il21^+/Cre^;Zfp36l1^fl/fl^* mice (Figure 2D) and with no changes in GC B cell numbers in mice lacking ZFP36L1 expression in their Tfh cells (Figure 2E). Following receipt of helper signals from Tfh cells, GC B cells move to the dark zone (DZ) of the GC where very rapid division occurs [1]. We subdivided GC B cells into light zone (LZ) or DZ phenotypes by flow cytometry but found no difference in the proportions of LZ and DZ GC B cells in *Il21^+/Cre^;Zfp36l1^fl/fl^* mice compared with control mice (Figure 2D,E). This suggests that in the absence of ZFP36L1, Tfh cells retain their ability to drive GC B cell proliferation. We confirmed these findings with confocal imaging analysis of iLNs where normal numbers and sizes of GCs were observed in mice with ZFP36L1‐deficient Tfh cells (Figure 2F,G). Additionally, the LZ:DZ ratio and the numbers of Tfh in each GC were also unaffected in *Il21^+/Cre^;Zfp36l1^fl/fl^* mice compared with control mice (Figure 2F,G).

### 2.3 Affinity Maturation Is Resilient to Loss of ZFP36L1 Expression in Tfh Cells

**FIGURE 3:**
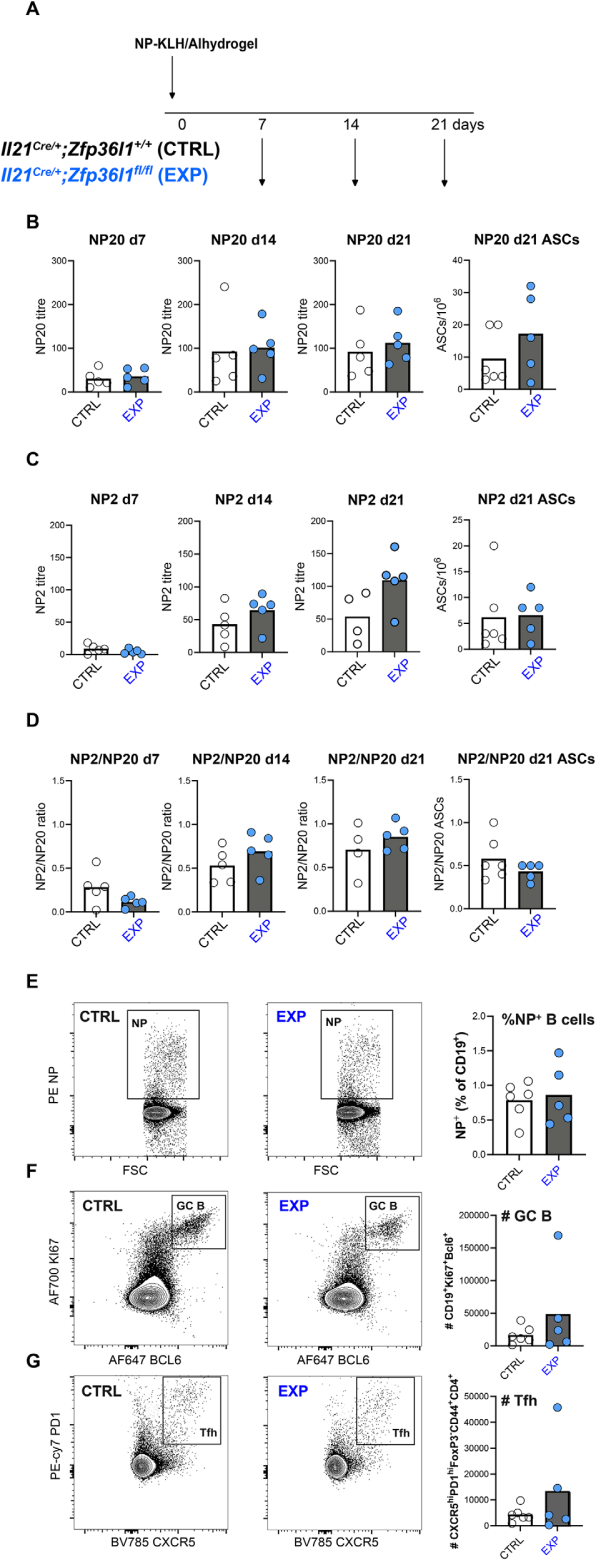
Affinity maturation is resilient to loss of ZFP36L1 expression in Tfh cells (A), Experimental scheme for (B–D). *Il21^+/Cre^;Zfp36l1^+/+^* (CTRL) and *Il21^+/Cre^;Zfp36l1^fl/fl^* (EXP) mice were immunized subcutaneously with NP‐KLH/Alhydrogel, and blood was taken on days 7,14, and 21 post‐immunization (p.i.), with bone marrow harvested on day 21 p.i. (B) Bar graphs showing titers of anti‐NP20 IgG1 (total affinity) on days 7, 14, and 21 p.i. and the number of ASCs on day 21 p.i. from CTRL (white circles) and EXP (blue circles) mice. (C) Bar graphs showing titers of anti‐NP2 IgG1 (high affinity) on days 7, 14, and 21 p.i. and ASCs on day 21 p.i. from CTRL (white circles) and EXP (blue circles) mice. (D) Bar graphs showing affinity maturation as determined by titers of NP2 relative to NP20 antibodies and numbers of bone marrow ASCs from CTRL (white circles) and EXP (blue circles) mice. (E) Representative flow cytometry plots showing the proportion of NP+ cells and bar graphs showing the proportion of NP+ cells amongst CD19+ B cells in draining iLN 21 d.p.i. in CTRL (white circles) and EXP (blue circles). (F) Representative flow cytometry plots showing the proportion of GC B cells and bar graphs showing the number of GC B cells in draining iLNs 21 d.p.i. in CTRL (white circles) and EXP (blue circles) mice. (G) Representative flow cytometry plots showing the proportion of Tfh cells and bar graphs showing the number of Tfh cells in draining iLNs 21 d.p.i. in CTRL (white circles) and EXP (blue circles) mice. Bar graphs show median values with each symbol representing an individual mouse. Data are representative of two independent experiments with 4–8 mice per group. A two‐tailed, unpaired Mann–Whitney *U* test was performed to determine statistical significance; statistics were omitted from graphs if the *p*‐value was not significant.

The hallmark of the GC response is affinity maturation of the antibody response [25]. This is dependent on Tfh cells providing helper signals to GC B cells in a competitive manner. It enables the evolution of the GC B cell response and selection of GC B cells with B cell receptors that bind the immunizing antigen effectively enough to present it to Tfh cells [1]. To test how ZFP36L1 expression in Tfh cells contributes to affinity maturation, we measured the antibody response to NP‐KLH over 3 weeks, allowing the evolution of the response to be characterized using ELISAs (for serum antibodies) and ELISpots (for antibody‐secreting cells) to measure total and high‐affinity antibodies to NP. Control (*Il21^+/Cre^;Zfp36l1^+/+^)* and experimental (*Il21^+/cre^;Zfp36l1^fl/fl^*) mice were immunized with NP‐KLH/Alhydrogel on both flanks, and blood was taken on days 7, 14, and 21 post‐immunization (p.i.; Figure 3A). In addition, bone marrow antibody‐secreting cells (ASCs) with total and high‐affinity NP binding capabilities were measured by ELISpot at day 21 p.i. There was no difference in the levels of total NP20‐binding antibodies and numbers of ASCs between *Il21^+/Cre^;Zfp36l1^fl/fl^* and control mice (Figure 3B). Loss of ZFP36L1 in Tfh cells also had no statistically significant impact on the generation of high‐affinity (NP2) binding antibodies, although there was a trend for increased titers in ZFP36L1‐deficient mice at day 21 p.i. (Figure 3C). We determined the degree of affinity maturation occurring within individual mice by calculating the ratio of NP2/NP20 titers. This showed that the lack of ZFP36L1 in Tfh cells had no detectable effect on affinity maturation (Figure 3D). There were also no perturbations in the proportions of NP^+^ B cells, and numbers of either GC B cells or Tfh cells at the day‐21 timepoint (Figure 3E–G).

## 3 Concluding Remarks

The provision of rapid but controlled help to B cells during the GC response is paramount to an effective immune response. It facilitates the generation of B cells capable of producing antibodies that bind with sufficient affinity, avidity, and selectivity for the immunizing antigen [20]. Tfh and B cell engagement in the GC occurs by entanglement, resulting in rapid surface display of CD40L (and presumably other helper factors) by Tfh cells [26]. Post‐transcriptional regulation of Tfh helper factors offers a mechanism to ensure this rapid but temporal response. By using a model system where we could specifically ablate ZFP36L1 in Tfh cells, we were able to determine the role of this RBP in Tfh cell function. However, despite the expression of ZFP36L1 protein in Tfh cells and evidence that *Zfp36l1* mRNA preferentially associates with Tfh cells after the Tfh/Th1 bifurcation point, there was no observable phenotype in *Il21^+/Cre^;Zfp36l1^fl/fl^* mice in terms of Tfh cell differentiation, GC responses, and affinity maturation. It is possible that absence of Zfp36l1 in Tfh cells does cause alterations in expression of cytokines and/or costimulatory molecules, but compensatory mechanisms are mounted that enable normal GC responses.

It is probable that other ZFP family members compensate for a lack of ZFP36L1 in Tfh cells, as has been seen in other T cell subsets. As described above, deletion of both ZFP36L1 and ZFP36L2 results in far greater production of IFN‐γ by CD8^+^ cells than observed by deletion of just a single ZFP family member. Likewise, ZFP36L1 restrains CD8^+^ T cell cytotoxic function, but it is only upon deletion of both ZFP36L1 and ZFP36L2 that this is revealed [10]. Also, mice with Treg cells deficient in ZFP36L1 show a milder phenotype than that of mice with a combined deletion of ZFP36L1 and ZFP36L2 [15].

The mechanisms controlling elicitation of Tfh cell help are likely to be multilayered, acting both pre‐ and post‐translationally on helper proteins. RNA‐binding proteins, such as the ZFP family, potentially provide a key layer of regulation and remain promising candidates for controlling the elicitation of Tfh cell help for the GC B cell. The complex redundancy that exists within the ZFP family of RBPs suggests that their function in Tfh cells will most likely be revealed by deleting multiple family members. Whilst this study shows that ZFP36L1 is dispensable for primary Tfh responses, it remains to be determined whether ZFP36L1 is important for the generation of the memory Tfh cell pool and recall responses.

## 4 Data Limitations and Perspectives

Here, we focused on the effect of deleting the RNA‐binding protein, ZFP36L1, from Tfh cells on a primary T cell‐dependent immune response. We compared the GC response mounted in ZFP36L1‐sufficient and deficient Tfh cells using *Il21*‐fatemapping to mark Tfh cells and their precursor populations. The lack of a measurable effect of ZFP36L1 deficiency on the GC response and affinity maturation may be masked by compensatory mechanisms that exist within the system. For example, expression of IL‐21 was not measured in this study. IL‐21 is the canonical cytokine associated with Tfh cell function. Future studies should address IL‐21 production in ZFP36L1‐deficient Tfh cells to address Tfh functionality in greater depth. ZFP36L1 is a member of a family of RNA‐binding proteins that may provide a compensatory role.

## 5 Materials and Methods

### 5.1 Mice

*II21^+/Cre^;Zfp36l1^fl/fl^* and *Il21^+/Cre^;Zfp36l1^+/+^* mice were bred and housed at the Babraham Institute Biological Support Unit. All mice were housed in individually ventilated cages, at an ambient temperature of 19–21°C, 52% relative humidity, and under pathogen‐free conditions. All mice used in experiments were between 8 and 12 weeks of age and a mix of sexes. All mouse work and experimentation were approved by the Babraham Institute Animal Welfare and Ethical Review Body and complied with all European Union and United Kingdom Home Office legislation and standards (PPL: P4D4AF812 & PP9973990).

### 5.2 Immunizations

Mice were immunized by subcutaneous injection of 50 µg NP‐KLH (N‐5060‐25, Biosearch Technologies) in a 1:1 ratio with 2% Alhydrogel (vac‐alu‐10, InvivoGen), 100 µL per flank.

### 5.3 Flow Cytometry

Cells were processed as previously described [20]. Gating strategies are shown in Figure S1. In brief, inguinal lymph nodes (iLNs) were pushed through a 30 µm cell strainer (1050552, CellTrics) to create a single cell suspension in FACS buffer (2% FBS (F9665, Sigma), in PBS with 0.05 mM EDTA (15575020, Invitrogen)). Cells were counted with CASY TT Cell Counter (OLS OMNI Life Science), and 2 × 10^6^ cells were plated per well in a v‐bottom 96‐well plate (611V96, Thermo Fisher Scientific). Cells were stained with surface antibodies for 2 h at 4°C, fixed with 2% formaldehyde (1.00496.0700, Sigma‐Aldrich) for 23 min at room temperature (RT), permeabilized with FoxP3 Permeabilization Buffer (00‐8333‐56, Invitrogen), and stained with intracellular antibodies overnight at 4°C. Antibodies are listed in Tables S1 and S2. For staining of ZFP36L1, cells were stimulated with PMA/Ionomycin (00‐4970‐93, Invitrogen) in complete RPMI (21875‐034, Gibco) supplemented with 10% (v/v) FBS (F9665, Sigma), 1% (v/v) penicillin‐streptomycin (15140‐122, Thermo Fisher Scientific), and 55 µM β‐mercaptoethanol (21985023, Thermo Fisher Scientific) for 3 h at 37°C. Cells were stained and fixed as above but with surface antibody incubation time reduced to 45 min.

### 5.4 Microscopy

iLNs were fixed with 4% PFA (J19943.K2, Thermo Fisher Scientific) for 2 h at 4°C, washed briefly with PBS and left overnight in 30% sucrose (w/v) (S/8600/60, Fisher Chemical) to cryopreserve. iLNs were embedded in O.C.T medium (AGR1180, Agar Scientific), frozen on dry ice and sectioned at 10 µm at −20°C with a Leica Cryostat (Leica CM3050S). For immunofluorescence staining, slides were dried under airflow for 10 min, rehydrated in PBS‐Tw20 (0.5% Tween‐20 (P1379, Sigma‐Aldrich) (v/v) in PBS) for 15 min and blocked with 10% normal donkey serum (D9663, Sigma‐Aldrich) (v/v) in 2% BSA (A7906, Sigma‐Aldrich in PBS for 45 min at RT. Slides were incubated with the primary antibodies listed in Table S3 for 1 h at room temperature and the secondary antibody listed in Table S3 for 30 min at room temperature. All antibodies were diluted in 1% BSA (w/v) in PBS‐Tw20. Slides were washed with PBS‐Tw20 for 5 min, three times between all staining and blocking steps. Finally, slides were washed briefly twice with PBS‐Tw20 and twice with PBS before mounting with Hydromount mounting medium (S6‐0106, Geneflow). Images were acquired on a Leica Stellaris 8 confocal microscope using a 20×/0.75 numerical aperture air lens. Every germinal center (GC) per LN was imaged at 512 × 512 resolution. Images were processed with ImageJ (v.2.14.0/1.54f) and quantitative analysis performed using a custom CellProfiler (v.4.2.4) pipeline. GCs were identified as CD35^+^ and/or Ki67^+^. Within the GC, the light zone was identified as CD35^+^ and the dark zone as CD35^−^Ki67^+^. Tfh cells were identified as PD1^+^ and their numbers quantified within each GC compartment using CellProfiler.

### 5.5 ELISAs

Flat‐bottom Nunc Maxisorb plates (456537, Thermo Fisher Scientific) were coated with 50 µL of either NP‐20‐BSA (10µg/ml, N5050G‐100, Biosearch Technologies) or NP‐2‐BSA (2µg/ml, N5050L‐100, Biosearch Technologies) diluted in PBS, overnight at 4°C. Plates were blocked with 200 µL of 2% BSA (A7906, Sigma‐Aldrich) (w/v) in PBS‐Tw20 (0.05% Tween‐20 (P1379, Sigma‐Aldrich) (v/v) in PBS) for 2 h at RT. Serum was serially diluted on the plate 1:3 from a starting concentration of 1:50 and incubated for 1 h at RT. For detection of serum antibodies, the plate was incubated with 50 µL anti‐mouse IgG1‐HRP (1/8000, ab97240, Abcam) for 1 h at RT, followed by 50 µL TMB substrate (421101, BioLegend) until an appropriate dynamic range was reached. The reaction was quenched by addition of 50 µL H_2_SO_4_ (2.5 N), and optical density measurements were taken at 450 nm on a PHERAstar FS plate reader (BMG Labtech).

### 5.6 ELlSpot

Ninety‐six‐well multiscreen HTS HA filter plates (MSHAS4510, Sigma‐Aldrich) were coated with 50 µL NP‐2‐BSA (2 µg/mL, N5050L‐100, Biosearch Technologies) or NP‐20‐BSA (10 µg/mL, N5050G‐100, Biosearch Technologies) overnight at 4°C. Plates were then incubated with 50 µL of complete RPMI (21875‐034, Gibco) supplemented with 10% (v/v) FBS (F9665, Sigma), 1% (v/v) Penicillin‐Streptomycin (15140‐122, Thermo Fisher Scientific), and 55 µM β‐mercaptoethanol (21985023, Thermo Fisher Scientific) for 1 h at RT. Bone marrow was extracted from the hind legs of the mice, and red blood cells were lysed by incubation with Ammonium‐Chloride red blood cell lysis buffer for 5 min. Bone marrow cells were resuspended in complete RPMI medium, and 2 × 10^6^ cells were plated in the first well, then serially diluted 1:2 down the plate and incubated overnight at 37°C. Plates were washed and then incubated with 50 µL anti‐mouse IgG1‐HRP (1/8000, ab97240, Abcam) for 1 h at RT and developed with 40 µL AEC chromogen substrate kit (AEC101‐1KT, Sigma‐Aldrich). Once wells were sufficiently developed, the plates were washed under running water and dried overnight in the dark before images were captured with an ImmunoSpot plate reader (Cellular Technology Ltd). Plates were washed between all steps with PBS‐Tw20 five times and dH_2_O three times.

### 5.7 Statistical Analysis

Data are representative of two independent experiments with 4–8 mice per group. Mice included in experiments were mixed sex and age‐matched as closely as possible. Comparisons between control and experimental groups were assessed by unpaired two‐tailed Mann–Whitney *U* test, two‐way ANOVA, or a Kruskal–Wallis test for multiple comparisons, with *p* < 0.05 considered statistically significant. All data points were included. Statistical analysis was performed in GraphPad Prism (v.9.4.1).

## Author Contributions

L.W. designed and performed experiments, analyzed data, constructed the figures, and co‐wrote the paper. H.A.C. designed and performed experiments, analyzed data, constructed the figures, and co‐wrote the paper. E.M.W. and S.W. designed and performed experiments. S.E.B. and M.T. provided mice and provided critical feedback. M.A.L. designed experiments and provided critical feedback. All authors contributed intellectually to the work and reviewed, edited, and approved the paper.

## Funding

This study was supported by funding from the Biotechnology and Biological Sciences Research Council (BBS/E/B/000C0427, Campus Capability Core Grant to the Babraham Institute), a UKRI Frontier award (EP/X022382/1) and a Lister Institute Prize Fellowship to M.A.L.

## Ethics Approval for Animal Studies Statement

All research complies with the relevant ethical regulations. All mouse experimentation and husbandry were performed in the United Kingdom with approval from the Babraham Institute Animal Welfare and Ethical Review Body and complied with European Union and UK Home Office legislation (Home Office License P4D4AF812, Project License PP9973990).

## Conflicts of Interest

Michelle A. Linterman reports funding from GSK outside of this work. The remaining authors declare no conflicts of interest.

## Supporting information




**Supporting File**: eji70260‐sup‐0001‐SuppMat.pdf.

## Data Availability

The data that support the findings of this study are openly available in E‐MTAB‐4388 at https://www.ebi.ac.uk/biostudies/arrayexpress.
