# Supplementary material for: Expression and Role of the RNA‐Binding Protein, ZFP36L1, in Mouse T Follicular Helper Cell Differentiation and Function
Source: Eur J Immunol. 2026 Aug 18;56(8):e70260. doi: 10.1002/eji.70260 (PMC13482495; doi:10.1002/eji.70260)
Supplement: Supplementary file 1 [file EJI-56-e70260-s001.pdf]

Supplementary Figure 1

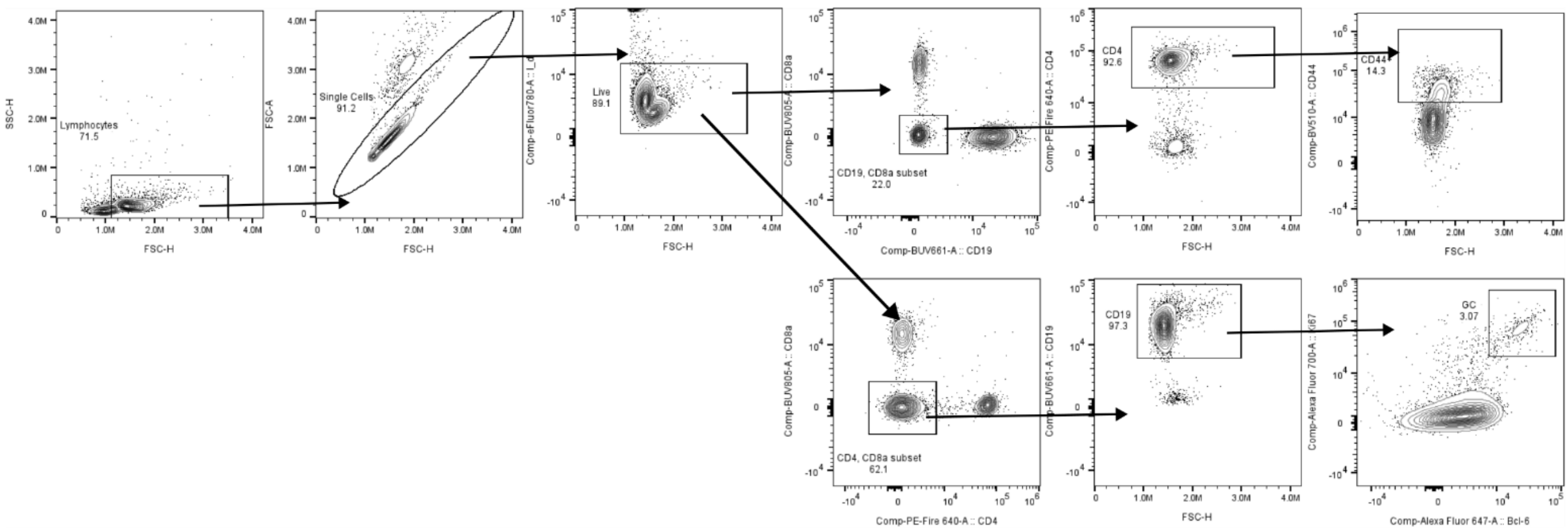

Supplementary Figure 1. Representative gating strategy for defining T and B lymphocyte subsets shown in Figures 1 & 2.

Supplementary Table 1: Flow Cytometry surface antibodies

Antigen	Fluorochrome	Clone	Vendor	Catalogue no.
CD90.2	BUV395	53-2.1	BD Bioscience	565257
CD19	BUV661	1D3	BD Bioscience	612971
CD8a	BUV805	53-6.7	BD Bioscience	612898
CD44	BV510	IM7	BioLegend	103044
CD44	BV711	IM7	BioLegend	103057
CD86	BV605	GL-1	BioLegend	105037
CD86	BV510	GL-1	BD Bioscience	563077
CXCR3	BV650	173	BioLegend	126531
CD138	BV711	281-2	BioLegend	142519
CXCR5	BV785	L138D7	BioLegend	145523
CD4	PE-Fire640	GK1.5	BioLegend	100481
PD1	PE-Cy7	29F.1A12	BioLegend	135215
CXCR4	APC	I.276F12	BioLegend	146508
IgD	SparkNIR685	11-26c-2a	BioLegend	405750
LiveDead	e780	-	Invitrogen	65-0865-14
B220	APCFire810	RA3-6B2	BioLegend	103278

Supplementary Table 2: Flow Cytometry intracellular antibodies

Antigen	Fluorochrome	Clone	Vendor	Catalogue no.
FoxP3	AF488	FJK-16s	Invitrogen	53-5773-82
Bcl6	AF647	A8	BioLegend	648306
Ki67	AF700	16A8	BioLegend	652419
FoxP3	eFluor450	FJK-16s	Invitrogen	48-5773-82
ZFP36L1	AF488	E6L6S	Cell Signalling Technology	30894S

Table 3: Confocal Microscopy antibodies

Antigen	Fluorochrome	Clone	Vendor	Catalogue no.
Ki67	eFluor450	SolA15	Invitrogen	48-5698-82
PD1	AF647	RMP1-30	BioLegend	109117
CD3	AF488	17A2	BioLegend	100210
CD35	biotin	8C12	BD Biosciences	553816
IgD	AF700	11-26c.2a	BioLegend	405730
Streptavidin	AF750	3 E4	Invitrogen	S21384
